# Promoting biodiversity in urban spaces: exploring the resilience of two wild plants, *Pancratium maritimum* L. and *Artemisia herba-alba* asso, in landscaping

**DOI:** 10.1186/s12870-025-07207-0

**Published:** 2025-09-09

**Authors:** Rewan A. Goda, Amal M. Fakhry, Laila M. Bidak, Soliman M. Toto

**Affiliations:** Department of Botany and Microbiology, Faculty of Science, Alexandria University, Alexandria, Egypt

**Keywords:** Drought stress mitigation, Sustainable landscaping, Irrigation regimes, Propagation techniques, Wildflower landscaping, Mediterranean flora, Water valorization

## Abstract

**Background:**

Because of their ecological, aesthetic, and beneficial characteristics, native desert plants are highly significant. They can also be utilized in landscape architecture, particularly in environments with harsh conditions. The present study aims to evaluate the potential utilization of the wild desert plants *Pancratium maritimum* L. (Amaryllidaceae) and *Artemisia herba-alba* Asso (Asteraceae) in sustainable landscape architecture strategies.

**Result:**

Pot experiments were conducted, including different water regimes and soil types. In the case of *P. maritimum*, all growth parameters showed significant differences (P˂ 0.001) among the water regime treatments, except ground cover. The same trend was notable between the two soil types, except for leaf area and maximum leaf length. Results recommended irrigating *P. maritimum* to 75% of field capacity when reached 30% in sandy soil (W2S5) due to its comparatively low water consumption (70 ± 12 L/ month/ m^2^) and relatively minimal effort, while maintaining the plant’s aesthetic value showing considerable values of growth parameters and being not significantly different from highest emmeans for each studied growth parameters. In the experiment of *A. herba-alba*, all growth parameters showed significant differences (P˂ 0.001) among the water regime treatments, with notable significant variations between the two soil types in all growth parameters. The results recommended irrigating *A. herba-alba* to 75% of field capacity when 35% is reached in its native soil (W2S2), as it shows the highest emmean value for all studied growth parameters. According to our findings, *P. maritimum* was successfully propagated by bulb, resulting in a 100% success rate. *A. herba-alba* demonstrated successful vegetative propagation by stem cuttings; both herbaceous and semi-woody, retaining its attractive appearance. Herbaceous cuttings of *A. herba-alba* are more successful in propagation, especially when using sandy soil in spring.

**Conclusion:**

*P. maritimum* and *A. herba-alba* had effective strategies to mitigate the adverse effects of drought stress. The present preliminary investigation’s findings may substantially improve water valorization by using native plants in landscaping, especially in arid and semi-arid regions.

**Graphical abstract:**

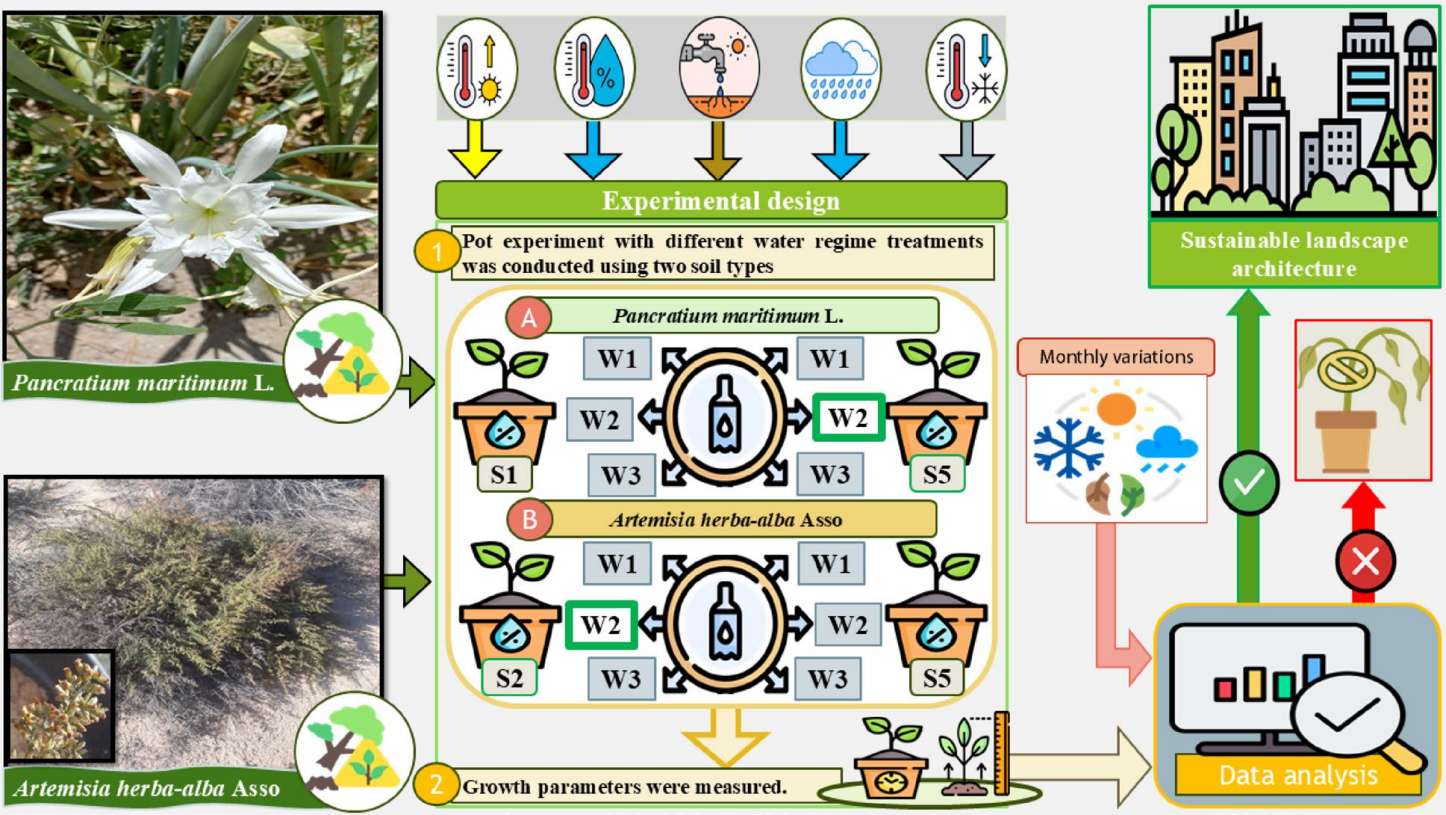

## Introduction

In many countries, including Egypt, where there is limited fresh water, the demand is rising, leading to more challenges. Urban greenery and landscaping activities in arid and semi-arid areas face significant difficulties due to limited water availability for landscape irrigation [1]. Therefore, innovative methods for reducing freshwater consumption in landscapes while preserving their aesthetic and functional characteristics should be developed [2]. A constant decline in plant biodiversity in natural habitats is perceived due to the increasing pressure of human impact on native species. The concept of incorporating wildflowers into landscapes is increasingly expected worldwide [1]. Urban landscaping can preserve biodiversity and provide a variety of ecological services. A varied native plant palette and the selection of drought-tolerant plants are two important landscaping decisions that can improve water conservation, boost pollinator resources, and reduce maintenance needs instantly [3]. Desert plants have the potential to be utilized in natural and cultural landscapes for ecological, aesthetic, beneficial, and economic reasons, particularly in harsh environments [4]. Recreation and coastal protection are just a few of the vital ecological services that desert coastal dune vegetation offers. However, it has also been shown to regulate greenhouse gas concentrations [5]. Human activities such as residential use and recreation, rapid urbanization, road development for cars and pedestrians, grazing, sand mining, and uncontrolled tourism negatively impact desert coastal areas and dunes. These actions disrupt the natural equilibrium of sand dunes and lower the number of dune plant species [6]. The utilization of native plant species in urban landscapes has gained significant attention in recent years [7]. Wild plants are often encouraged for gardening, biodiversity conservation, and ecological restoration [8]. Furthermore, conservation landscaping can contribute to environmental sustainability by reducing water use and creating biologically diverse habitats, aiding in the conservation of native species [7]. Moreover, native wild plants are more promising compared to exotic plant species as they readily adapt to the local environment [9]. Two wild plants, *Pancratium maritimum* L. (Amaryllidaceae) and *Artemisia herba-alba* Asso (Asteraceae), were selected for the present study to examine their potential use in urban landscaping. Both species are native to the flora of Egypt, with their native range being the Mediterranean. *A. herba-alba* is a greyish perennial dwarf plant also known as desert wormwood and Sheeh in Arabic [10]. The plant is heavily harvested from the wild and is currently regarded as a threatened species due to its many medicinal uses. Its leaves emit a strong scent and are adorned with tiny granular hairs that reflect sunlight, giving them a grayish appearance. The plant blooms from September to December with yellow flowers. It is also recognized for its ability to adapt to drought stress [11]. *P. maritimum* is a perennial geophyte known as sea daffodil, sea lily, or sand lily. It grows in the wild and is widespread along the Mediterranean coastal areas. Over-tourism and human activities may limit the expansion of *P. maritimum* populations. This plant blooms during the dry summer, producing large inflorescences of white flowers that have exceptional beauty and scent, conferring significant ornamental value. The buds on the subterranean perennial organ are protected beneath the soil’s surface, and there is a dormant phase between periods of growth [12]. By identifying the potential use of native wild plants in sustainable landscape architecture practices through the application of various irrigation treatments, this study may provide guidance that will assist in the conservation of plant species and the development of diverse landscapes, especially in peri-urban, restoration, or coastal xeriscaping projects.

## Materials and methods

### Plant material

Two disturbed locations at the Western Mediterranean Coastal Region in Egypt were chosen to collect plant materials, 30.981566 N, 29.577671 E, Sidi Kirayr 41 Km west of Alexandria 26 m MSL, for *Pancratium maritimum* L., 30.818549 N, 29.073086 E, El-Alamein 95 Km west of Alexandria 12 m MSL, for *Artemisia herba-alba* Asso in the early winter of 2023.

Plants were collected under the relevant national regulations and the international guidelines of the IUCN [13] and the Convention on the Trade in Endangered Species of Wild Fauna and Flora (CITES) [14]. Permission to collect the plant specimens of the species under investigation for scientific purposes was obtained from the Department of Botany and Microbiology at the Faculty of Science, Alexandria University.

Plants were identified by Amal M. Fakhry, Professor of biodiversity, Department of Botany and Microbiology, Faculty of Science, Alexandria University. Voucher specimens were deposited in the Herbarium of Alexandria University (ALEX) at the Faculty of Science, deposition numbers ALEX 4119 for *P. maritimum* and ALEX 41120 for *A. herba-alba*.

Seedlings selection was based on their size and ease of harvesting to minimize environmental impact and damage. The collected seedlings were transferred to the Botanic Garden of the Faculty of Science, Alexandria University, and temporarily maintained in plastic cups containing soil from their original collection sites. After a two-week acclimatization period, each seedling was transplanted into an individual pot. To promote proper establishment and minimize transplant shock, the plants were irrigated regularly using tap water. For each plant species, 15 pots were prepared, corresponding to five replicates for each treatment. Of these, three replicates were specifically used for monitoring and measuring plant growth.

### Growing conditions, irrigation treatments, and experimental design

The experiments were conducted for twelve months using two plant species, as they were set up as a 2 × 3 factorial design with two independent variables: soil type and water regime. The first factor, soil type, included two variants: native field soil collected from the natural habitats of the target species (designated as S1 for *P. maritimum* and S2 for *A. herba-alba*) and urban sandy soil (S5), commonly utilized in landscaping applications. The second factor, water regime, was tested at three levels: for *P. maritimum*, the regimes included 50–75% field capacity (FC; W1), 30–75% FC (W2), and 30–50% FC (W3); for *A. herba-alba*, the levels were 50–75% FC (W1), 35–75% FC (W2), and 35–50% FC (W3). To ensure uniform initial moisture conditions, all pots were watered with tap water (electrical conductivity of 0.69 dS m⁻¹) until the soil was fully saturated. Subsequently, based on the assigned treatment and plant species, the pots were weighed periodically and irrigated once the soil moisture content declined to 50%, 35%, or 30% of FC, as specified for each treatment. Each water regime had three replicates.

Before applying irrigation treatments, soil samples from each type of soil were examined for their chemical and physical characteristics according to Qazi [15]. The field capacity (FC) and bulk density for every type of soil were calculated before the experiments started. Tap water was used for watering three pots with a soil mass ranging from 5.2 to 6.8 kg to maintain a constant volume (21 cm top diameter, 18 cm base diameter, and 15.5 cm height). After covering the pots’ surface, the pots were left for 48 h to drain under undisturbed conditions. Upon completion of the drainage phase, three soil subsamples (70 g each) were collected from the central region of each pot. The wet weight of each subsample (denoted as A) was recorded prior to oven-drying at 90 °C for 72 h. Following the drying process, the samples were reweighed to determine the dry weight (denoted as B). The formula WHC = [(A – B) × 100]/B was then used to determine the water holding capacity (WHC). That portion of the WHC was used to estimate the soil field capacity at 75%, 50%, 35%, and 30%, according to Mahajan et al. [16]; Signorelli et al. [17]. The core method (volumetric cylinder method) was used to measure bulk density [18]. The volume of the monthly water used per kilogram of soil (L/month/kg) was multiplied by the bulk density of the soil (kg/m^3^), irrigated area (1m^2^) and the depth of the soil (m) to determine the monthly water need per square meter of irrigated area (L/month/m^2^) [19].

### Plant growth variables

In the case of *P. maritimum*, growth was assessed using the maximum leaf length (the longest leaf of the plant) (cm), number of leaves, ground cover (cm²), and leaf area (cm²) as growth indicators. For *A. herba-alba*, plant height (cm) and ground cover (cm²) were employed as the growth variables to evaluate vegetative growth. The plant ground cover and leaf area were assessed during the experiment by taking images of each experimental pot using the Samsung Galaxy A32 mobile device from a fixed distance (79 cm). The image-processing program ImageJ ver. 1.53t was used to calculate the surface area of plant ground cover (cm^2^) and leaf area (cm^2^) [20, 21].

### Propagation

The vegetative propagation of *P. maritimum* was carried out by bulb in January 2023, collected from Egypt’s Western Mediterranean Coastal region at 30.937° N, 29.546° E, 26 m MSL. Vegetative propagation of *A. herba-alba* was performed using stem cuttings of both herbaceous and semi-woody varieties, with transplants collected in January 2023 from its natural habitat in Egypt’s Western Mediterranean Coastal region at 30.843° N, 29.142° E, 12 m MSL. These transplants were domesticated for one year prior to propagation. The experiment utilized sandy soil. To evaluate the impact of seasonal variations on vegetative propagation, two trials were conducted in the winter and spring of 2024.

### Statistical analysis

The statistical analyses were conducted using R v4.3.1 with RStudio software [22]. Before selecting the appropriate statistical method, the data were assessed for normality using the Shapiro–Wilk test and for homogeneity of variances using Levene’s test. The assumptions of normality and homogeneity of variances were not met with either experiment. All results are expressed as mean ± standard deviation (SD) based on three replicates. A generalized linear mixed model (GLMM) was used to evaluate significant differences among soil type and soil moisture (water regime) factors. An analysis of deviance table (Type II Wald F tests with Kenward-Roger degrees of freedom) was generated using the ‘Anova’ function from the ‘car’ package to assess the main effects of soil type, soil moisture, and their interaction. For pairwise post hoc multiple comparisons, the ‘emmeans’ package [23] was utilized, applying Tukey-adjusted comparisons for p-value adjustments.

## Results

### Physical and chemical properties of substrate

Physical and Chemical analysis of the soils used in this study is presented in Table 1. The results showed that the electric conductivity (EC) of native soil for *Pancratium maritimum* (S1) was considerably lower than urban sandy soil (0.64 and 1.46 ds/m respectively), while EC of native soil (S2) for *Artemisia herba-alba* was nearly the same as the urban sandy soil (S5) used in the experiment (1.51 and 1.46 ds/m respectively). The soil analysis also showed that the soluble cations (Na^+^, Mg^2+^ and Ca^2+^) and the soluble anions (Cl^−^, HCO_3_^−^ and SO_4_^2−^) of native soil for *P. maritimum* (S1) were considerably lower than urban sandy soil (S5), except for K^+^ and HCO_3_^−^ which were higher in native soil (S1). Conversely, the soluble cations (Na^+^, K^+^ and Ca^2+^) and the soluble anions (Cl^−^, HCO_3_^−^ and SO_4_^2−^) for native soil of *A. herba-alba* (S2) were considerably higher than urban sandy soil (S5), except for Mg^2+^ and SO_4_^2−^ which were lower than that of urban sandy soil (S5). Although most soils used in our experiments had sandy texture, the native soils of *P. maritimum* and *A. herba-alba* had significantly higher clay and silt fractions than the urban sandy soils.

**Table 1:** Characteristics of soils used in the study

Soil sample	pH	EC dS/m	Soluble Salts (meq/l)								TDS ppm	O.M %	CaCO_3_ %	Particle Size Distribution [%]			Texture
			Ca^++^	Mg^++^	Na^+^	K^+^	CO_3_^−^	HCO_3_^−^	Cl^−^	SO_4_^−−^				Sand	Silt	Clay	
S1	8.19	0.64	2.4	1	3.43	1.3	0	2.4	2.4	3.33	410	0.7	9.7	74	20	16	Sand
S2	8.24	1.51	6.8	4.9	7.5	2.1	0	3	6.76	11.6	966	0.6	6.8	74	12	14	Sand
S5	8.44	1.46	6.1	5.6	7.4	0.9	0	2	4.3	13.7	934	0.3	2.8	97	1	2	Sand

### Growth parameters under different water regime irrigation treatments

#### *Pancratium maritimum*

Throughout the experimental period, all evaluated growth parameters (ground cover, leaf area, maximum leaf length, and number of leaves) demonstrated a distinct temporal pattern. Mean values increased progressively from the onset of the experiment up to April, followed by a marked decline from May to June, then near or complete disappearance during the dormant stage between months July and September. A subsequent recovery and renewed increase in all growth parameters were observed post-dormancy, beginning from October onward (Table 2). In native field soil (S1), water regime W2 resulted in the highest mean values for ground cover, number of leaves, and maximum leaf length throughout the study period, followed by W1, whereas W3 exhibited the lowest mean values. Notably, leaf area under W1 in S1 showed the highest mean values, followed by W3, with W2 showing the lowest. In sandy soil (S5), W2 again demonstrated the highest mean values for ground cover over the study period, followed by W1, with W3 showing the lowest. However, for leaf area and maximum leaf length, W1 yielded the highest mean values, followed by W2, while W3 remained the lowest. Regarding the number of leaves in S5, W2 produced the highest mean values, followed by W3, with W1 showing the lowest.

**Table 2:** Means and standard deviations of the studied growth parameters of *Pancratium maritimum* seedlings under different types of soils and water regimes. The values are means ± sd

Growth parameters	Soil type	Water regime	Months								
			January	February	March	April	May	June	October	November	December
**Ground cover (cm** ^**2**^ **)**	**S1**	**W1**	82.4 ± 4.5	133 ± 25.5	147 ± 35.7	160.3 ± 40.7	92.8 ± 43.1	12.2 ± 5	14.5 ± 11.4	32 ± 25.7	64 ± 61.5
		**W2**	82.4 ± 4.5	160 ± 99.9	175.1 ± 114.9	150.6 ± 113.8	92 ± 87.9	7.8 ± 8.7	23.2 ± 12.7	47.3 ± 21.8	84.3 ± 41.4
		**W3**	82.4 ± 4.5	106 ± 40.2	109.5 ± 47.1	92.5 ± 29.3	73.4 ± 19.2	12 ± 5.2	11.3 ± 7.7	28.2 ± 13.8	53.2 ± 27.3
	**S5**	**W1**	82.4 ± 4.5	80.3 ± 10.2	92.3 ± 7.8	85 ± 8.2	85.8 ± 38.5	5.3 ± 4.9	6.8 ± 6.2	22.1 ± 12.9	45.7 ± 12.3
		**W2**	82.4 ± 4.5	103.8 ± 50.7	104.1 ± 54.5	83.9 ± 29.2	68.9 ± 44.7	1.2	5.6 ± 4.9	22.3 ± 15.9	48.7 ± 37.5
		**W3**	82.4 ± 4.5	88 ± 9	105.2 ± 28	65.7 ± 15.3	45.7 ± 20	3.3 ± 3.3	4.8 ± 1.3	15.5 ± 5.9	45.6 ± 6.3
**Leaf area (cm** ^**2**^ **)**	**S1**	**W1**	2.0 ± 8	15.1 ± 4.6	16.6 ± 7.7	20.5 ± 11.9	13 ± 6.7	2.8 ± 0.8	4.8 ± 3.3	7.3 ± 6	10.8 ± 11.3
		**W2**	2.0 ± 8	13 ± 1.6	13.4 ± 3.8	13.6 ± 5.1	12 ± 8.2	2.3 ± 0.9	4 ± 0.9	5.9 ± 0.6	8.1 ± 2.5
		**W3**	2.0 ± 8	11.1 ± 0.4	12.3 ± 0.9	11 ± 1	11.7 ± 3.5	5 ± 3.3	5.3 ± 3.4	6.8 ± 1.3	7.5 ± 2.5
	**S5**	**W1**	2.0 ± 8	10 ± 1.1	11.3 ± 2.9	14.2 ± 1.9	19.6 ± 0.5	3.1 ± 1.8	3.7 ± 2	7.1 ± 1.4	8.9 ± 0.4
		**W2**	2.0 ± 8	12.3 ± 1.8	12.5 ± 5	12.5 ± 4.1	17.8 ± 5.7	1.2	3 ± 1.3	6.1 ± 3.9	9.2 ± 2.1
		**W3**	2.0 ± 8	8.3 ± 0.3	9.5 ± 0.5	7.4 ± 0.1	8.4 ± 3	3.3 ± 3.3	2 ± 0.7	3.2 ± 1.1	6
**Number of leaves**	**S1**	**W1**	5.3 ± 0.4	11.4 ± 2.4	12.3 ± 3.2	10.3 ± 3.5	10.3 ± 1.5	4.3 ± 0.6	2.3 ± 1.7	4.5 ± 2.2	9 ± 2.1
		**W2**	5.3 ± 0.4	13.1 ± 4.2	14.7 ± 6.1	10.3 ± 5.7	8 ± 3.5	2.7 ± 2.1	6.2 ± 2.8	8.8 ± 3.8	11 ± 5.6
		**W3**	5.3 ± 0.4	10.3 ± 1.2	11 ± 5.2	11 ± 5.6	8.3 ± 3.8	2.7 ± 1.2	2.1 ± 1	5 ± 3.3	9 ± 5
	**S5**	W1	5.3 ± 0.4	6.1 ± 1.3	7 ± 1.4	6.5 ± 0.7	5	1.5 ± 0.7	1.7 ± 0.9	4.5	9
		**W2**	5.3 ± 0.4	9.1 ± 1.1	10.3 ± 5.1	9 ± 4	6.7 ± 3.5	1	1.8 ± 1.1	4.2 ± 2.3	8 ± 2.3
		W3	5.3 ± 0.4	10 ± 2.2	12 ± 2.8	11 ± 1.4	7.5 ± 0.7	1	2.3 ± 0.5	6 ± 1.4	10 ± 4.2
**Maximum leaf length (cm)**	**S1**	**W1**	22.1 ± 0.5	30 ± 4	32.7 ± 4.8	31.7 ± 4.4	28.3 ± 5.5	13 ± 3.1	11.8 ± 5.7	15.9 ± 6.1	21.5 ± 6.2
		**W2**	22.1 ± 0.5	28.3 ± 2.5	30.3 ± 4	29.1 ± 4	27.8 ± 4.4	17.2 ± 1.5	14.6 ± 1.5	19.8 ± 0.8	21.8 ± 0.6
		**W3**	22.1 ± 0.5	25 ± 0.4	28.8 ± 0.5	30.7 ± 2.8	27.1 ± 3.2	15.7 ± 2.5	10 ± 1	17.2 ± 1.5	19.5 ± 2
	**S5**	**W1**	22.1 ± 0.5	29 ± 0.9	30.9 ± 0.4	30.8 ± 0.4	31.7 ± 0.4	16.3 ± 10.2	14.2 ± 0.7	17.7 ± 2.5	22.8 ± 0.5
		**W2**	22.1 ± 0.5	28.4 ± 1.1	30.9 ± 1.9	30.8 ± 2	29.2 ± 4.8	6	15.7 ± 2.5	16.9 ± 7	25.5 ± 4.9
		**W3**	22.1 ± 0.5	24.8 ± 0.3	26.1 ± 1.3	27.4 ± 0.9	25.8 ± 0.4	12.7 ± 3.8	9.4 ± 1	13.9 ± 1.7	19.5 ± 0.7

**W1**, 50–75% of field capacity; **W2**, 30–75% of field capacity; **W3**, 30–50% of field capacity; **S1**, native soil; **S5**, sandy soil

**Fig. 1:**
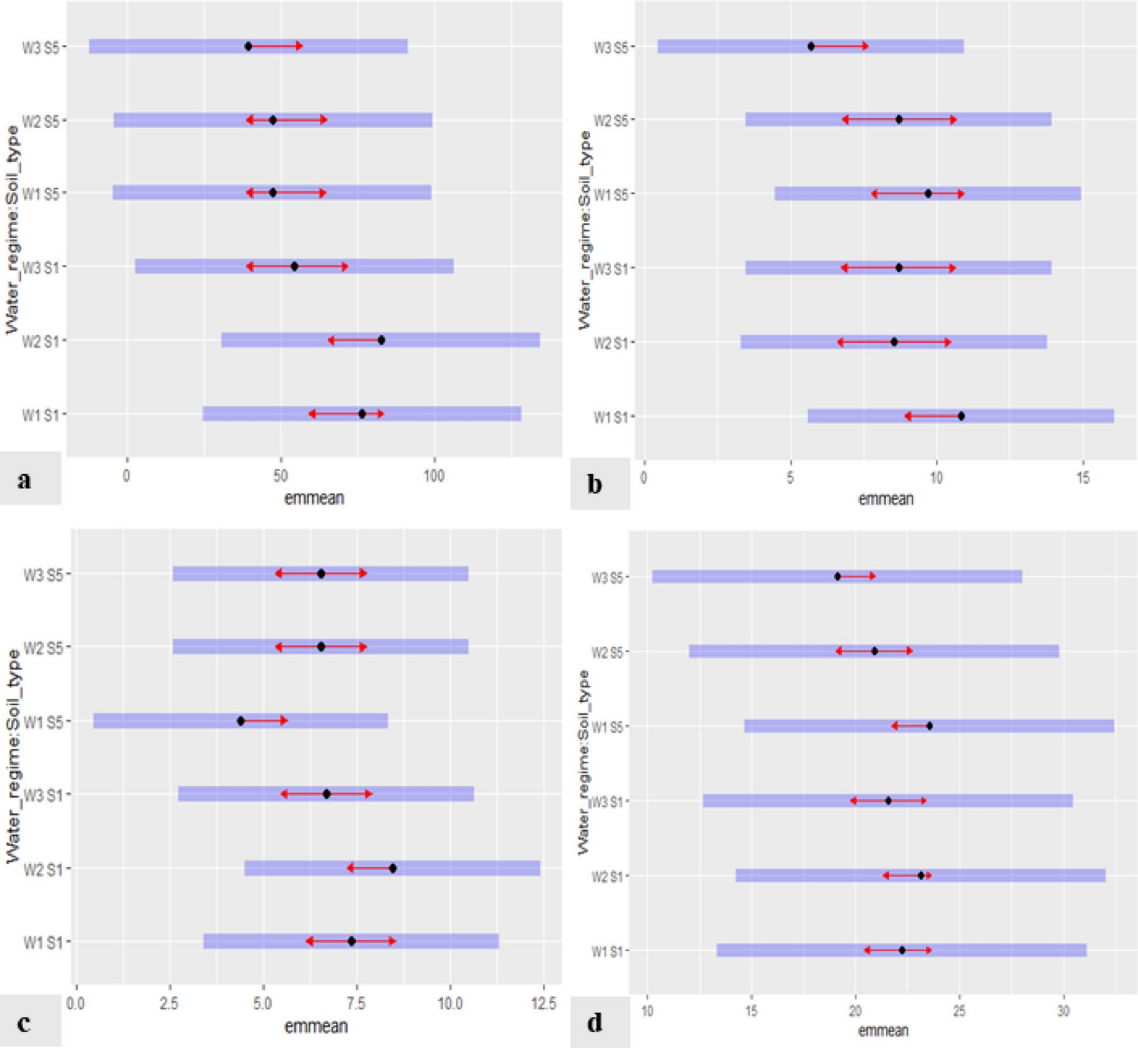
Estimated marginal means (emmean) of different water regimes/soil type experiments for growth parameters of *Pancratium maritimum*: ground cover **(a)**, leaf area **(b)**, number of leaves **(c)**, and maximum leaf length **(d)**. (The blue bars are confidence intervals for the estimated marginal means, and the red arrows are for the comparisons among them)

Post-hoc Tukey test with the estimated marginal means (emmeans) function was performed for pairwise post hoc multiple comparisons. Estimated marginal means of different water regime/soil type experiments for growth parameters of *P. maritimum* are presented in Fig. 1. The blue bars are confidence intervals for the emmean, and the red arrows are for the comparisons among them. If an arrow from one mean overlaps an arrow from another group, the difference is not “significant” based on the adjusted setting (which defaults to “Tukey”) and the value of alpha (which defaults to 0.05). The results showed that W2S1 has the highest emmean values for ground cover and number of leaves. The highest emmean values of leaf area were attained in W1S1, while the maximum leaf length attained the highest emmean values in W1S5. Statistical analysis indicated that the treatments (W1S5, W2S1, W1S1, and W2S5) are not significantly different. On the other hand, W3S5 showed the least emmean values for all studied growth parameters except for the number of leaves, which showed the least emmean values in W1S5. (Fig. 1).

#### *Artemisia herba-alba*

During the experimental period, ground cover exhibited a fluctuating trend, with an initial increase in mean values, followed by a decline, and a subsequent increase toward the end of the study. In contrast, plant height demonstrated a consistent increase in mean values throughout the observation period (Table 3). Under native field soil conditions (S2), water regime W2 resulted in the highest mean values for both ground cover and plant height, followed by W1, whereas the lowest values were recorded under W3. A similar pattern was observed in sandy soil (S5) for ground cover, where W2 maintained nearly the highest mean values, followed by W1, and the lowest under W3. Interestingly, for plant height in sandy soil (S5), W1 treatment produced the highest mean values, followed by W2, while W3 consistently resulted in the lowest values across the study period.

**Table 3:** Means and standard deviations of the studied growth parameters of *Artemisia herba-alba* seedlings under different types of soils and water regimes. The values are means ± sd

Growth parameters	Soil types	Water regime	Months											
			January	February	March	April	May	June	July	August	September	October	November	December
**Ground cover (cm** ^**2**^ **)**	**S2**	**W1**	2.7 ± 0.7	11.3 ± 2.2	12.5 ± 3.1	19.6 ± 9.4	70.4 ± 34.8	107.6 ± 65.2	112.2 ± 58.4	88.3 ± 44.3	87.7 ± 45.2	97.2 ± 52.7	107.5 ± 45.6	110.5 ± 40.1
		**W2**	2.7 ± 0.7	15.2 ± 10	16 ± 12	19.7 ± 11.9	136.9 ± 82.3	205.6 ± 86.2	231.4 ± 68.3	157 ± 46.3	122.1 ± 7.5	132.6 ± 21.7	148.5 ± 24.3	150.2 ± 20.8
		**W3**	2.7 ± 0.7	11 ± 4.4	12 ± 5.1	14.3 ± 12.3	82.6 ± 55.3	105 ± 61.5	108 ± 60.1	88.6 ± 42.5	83.9 ± 43.1	90.9 ± 55.3	100.5 ± 49.6	105.3 ± 35.7
	**S5**	**W1**	2.7 ± 0.7	4.3 ± 2.1	5.6 ± 4.6	6.6 ± 4.4	22.8 ± 17.3	39.4 ± 23.7	53.6 ± 31.9	50 ± 29.6	47.2 ± 24.2	52.4 ± 26.1	64.2 ± 24.6	65.8 ± 16.7
		**W2**	2.7 ± 0.7	10.4 ± 3.6	11.9 ± 5.7	14.5 ± 4.8	46.9 ± 4.4	65.7 ± 7.3	73.8 ± 12	58.9 ± 7.9	54.2 ± 12.2	65.2 ± 20.1	67.9 ± 19.4	69.9 ± 29.1
		**W3**	2.7 ± 0.7	3.2 ± 1.7	4.2 ± 3.7	2.6 ± 3.5	21.3	36.7 ± 0.1	33.9 ± 0.1	27.2 ± 0.1	28.1 ± 0.2	33.4 ± 0.1	44.3 ± 0.1	46.1 ± 0.5
**Plant height (cm)**	**S2**	**W1**	1 ± 0.1	2.8 ± 0.2	3.8 ± 0.3	7.9 ± 0.7	16.1 ± 2.1	19.9 ± 6.1	21.4 ± 4.6	21.4 ± 4.1	21.5 ± 4.3	21 ± 4	20.9 ± 4.4	15.7 ± 2.5
		**W2**	1 ± 0.1	4.1 ± 2	5.5 ± 3.9	12.2 ± 4.8	22.6 ± 2.5	25.2 ± 1.1	25.3 ± 0.5	25.1 ± 1.3	24.5 ± 2.9	24.1 ± 2.6	24.1 ± 2.8	21 ± 5
		**W3**	1 ± 0.1	4.4 ± 0.5	5.6 ± 1.9	9.9 ± 6.2	16.4 ± 9.8	16.8 ± 2.9	16.1 ± 2.3	16.2 ± 2.4	17 ± 3.2	16.6 ± 3.2	16.7 ± 3.2	21.9 ± 4.5
	**S5**	**W1**	1 ± 0.1	2 ± 0.3	2.2 ± 1.9	3.9 ± 3	10.5 ± 7.9	17.1 ± 7.9	19.6 ± 5.6	20 ± 4.7	20.3 ± 4.5	20.5 ± 4.5	20.4 ± 4	21.7 ± 2.3
		**W2**	1 ± 0.1	3.2 ± 1.4	4.6 ± 2.8	7.5 ± 3	14.1 ± 3	19 ± 3.9	19.8 ± 4.6	19.8 ± 4.4	19.3 ± 5.3	19.9 ± 4.3	18.9 ± 5.4	20 ± 6.6
		**W3**	1 ± 0.1	1.2 ± 0.2	1.4 ± 0.8	2 ± 1.1	7.4 ± 0.1	11.5 ± 0.5	12.1 ± 0.9	12.8 ± 1	12.8 ± 0.3	12.5 ± 0.3	12.8 ± 0.3	18.5 ± 0.1

**W1**, 50–75% of field capacity; **W2**, 35–75% of field capacity; **W3**, 35–50% of field capacity; **S2**, native soil; **S5**, urban soil

**Fig. 2:**
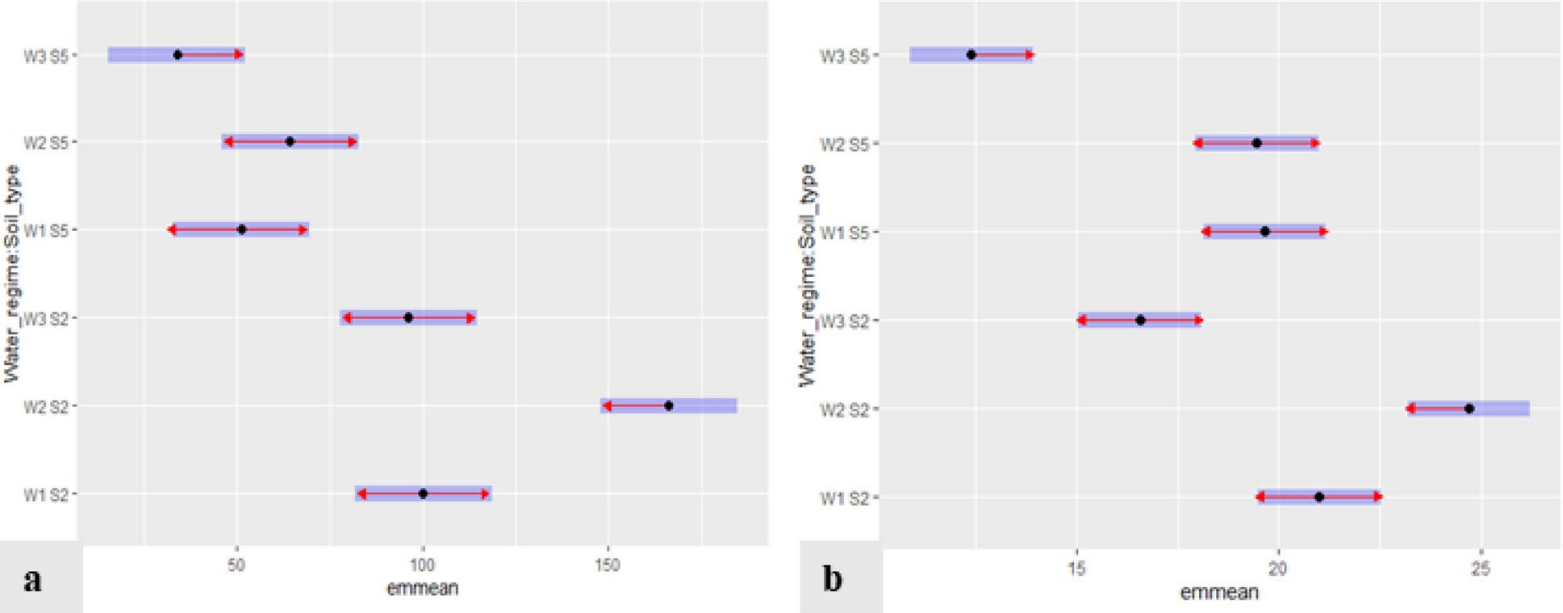
Estimated marginal means (emmean) of different water regimes/soil type experiments for growth parameters of *Artemisia herba-alba*: ground cover **(a)** and plant height **(b)**. (The blue bars are confidence intervals for the estimated marginal means, and the red arrows are for the comparisons among them.)

The Post-hoc Tukey test with the estimated marginal means (emmeans) function was performed for pairwise post hoc multiple comparisons. Estimated marginal means of different water regime/soil type experiments for growth parameters of *A. herba-alba* are presented in Fig. 2. The blue bars are confidence intervals for the emmean, and the red arrows are for the comparisons among them. If an arrow from one mean overlaps an arrow from another group, the difference is not “significant” based on the adjusted setting (which defaults to “Tukey”) and the value of alpha (which defaults to 0.05). The results showed that the highest emmean values for the tested growth parameters, ground cover and plant height, were obtained in the W2S2 treatment. The lowest values for the two tested growth parameters were obtained in the W3S5 treatment (Fig. 2).

#### Data analysis

All data sets were analyzed using a generalized linear Mixed model. The P-values calculated through the analysis of variance (ANOVA) indicate significant differences among the three levels of water regimes for all the studied growth parameters except for the ground cover of *P. maritimum* (Table 4). Significant differences are also noticed among the two soil types for all growth parameters except for leaf area and maximum leaf length of *P. maritimum*. Additionally, the soil type × water regime interaction has insignificant effects on the growth parameters of *P. maritimum* while having significant effects on the growth parameters of *A*. *herba-alba*.

**Table 4:** Analysis of deviance table (Type II Wald F tests with Kenward-Roger df) using generalized linear mixed model

Plant species	Factor	Response variable	F value	D.f.	Df.res	*P* value
***Pancratium maritimum***	Water regime	Ground cover	2.5967	2	97	**0.0796917**
		Leaf area	5.2633	2	97	0.006759 **
		Number of leaves	3.6615	2	97	0.0293078 *
		Maximum leaf length	4.2138	2	97	0.01759 *
	Soil type	Ground cover	14.5488	1	97	0.0002402 ***
		Leaf area	2.8928	1	97	**0.092182**
		Number of leaves	11.8049	1	97	0.0008711 ***
		Maximum leaf length	2.3625	1	97	**0.12754**
	Water regime: Soil type	Ground cover	0.7506	2	97	**0.4748**
		Leaf area	1.4078	2	97	**0.24963**
		Number of leaves	2.8297	2	97	**0.0639131**
		Maximum leaf length	2.8471	2	97	**0.06287**
***Artemisia herba-alba***	Water regime	Ground cover	18.9482	2	97	1.131e-07 ***
		Plant height	54.4737	2	97	< 2.2e-16 ***
	Soil type	Ground cover	102.343	1	97	< 2.2e-16 ***
		Plant height	33.129	1	97	1.009e-07 ***
	Water regime: Soil type	Ground cover	5.1433	2	97	0.007533 **
		Plant height	3.4667	2	97	0.03514 *

**Significance codes**: 0 ‘***’ 0.001 ‘**’ 0.01 ‘*’ 0.05 ‘.’ 0.1 ‘ ’ 1

#### Water consumption

Table 5 shows the monthly water needs per square meter of irrigated area (L/month/m^2^). In case of *P. maritimum*, results indicated that the lowest irrigation water used (44 ± 5 L/month/m^2^) was recorded for urban sandy soil in water regime W3, followed by native soil in water regime W3 (69 ± 6 L/month/m^2^) and urban sandy soil in water regime W2 (70 ± 12 L/month/m^2^). In case of *A. herba-alba*, the results indicated that the amount of irrigation water applied under each water regime treatment was nearly identical for both soil types, with the native soil (S4) consistently consuming slightly more water than the urban sandy soil (S5). The lowest irrigation water used (65 ± 4 and 66 ± 4 L/month/m^2^) was recorded for water regime W3, followed by water regime W2 (87 ± 10 and 88 ± 10 L/month/m^2^) then water regime W1 (109 ± 6 and 110 ± 6 L/month/m^2^).

**Table 5:** Irrigation water requirements for 1 m^2^ of land (L/month/m^2^) for the different soil types and different water regimes per month for the studied species. The values are means ± sd

Plant species	Soil type	Water regime	Amount of water (L/month/m^2^)
*Pancratium maritimum*	**S1**	W1	121 ± 7
	**S1**	W2	94 ± 13
	**S1**	W3	69 ± 6
	**S5**	W1	109 ± 6
	**S5**	W2	70 ± 12
	**S5**	W3	44 ± 5
Abbreviations: W1, 50–75% of field capacity; W2, 30–75% of field capacity; W3, 30–50% of field capacity; S1, native soil; S5, urban sandy soil. Soil layer depth = 0.155 m			
*Artemisia herba-alba*	**S2**	W1	110 ± 6
	**S2**	W2	88 ± 10
	**S2**	W3	66 ± 4
	**S5**	W1	109 ± 6
	**S5**	W2	87 ± 10
	**S5**	W3	65 ± 4
Abbreviations: W1, 50–75% of field capacity; W2, 35–75% of field capacity; W3, 35–50% of field capacity; S2, native soil; S5, urban sandy soil. Soil layer depth = 0.155 m			

#### Propagation

**Fig. 3:**
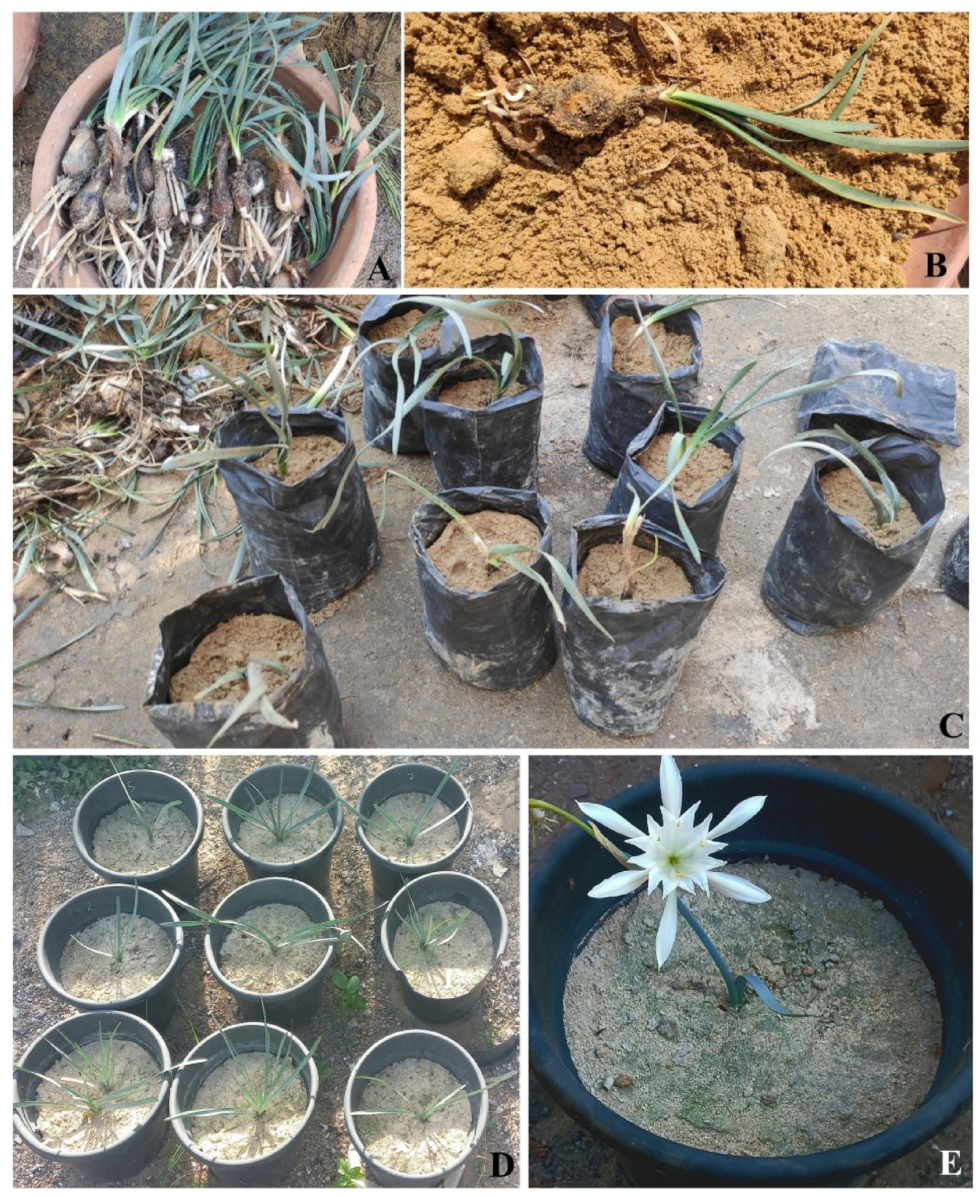
Propagation of *Pancratium maritimum*. The selected bulbs **(A)** and **(B)**, Bulbs planted in a plastic bag **(C)**, successful growth after a year **(D)**, and inflorescence **(E)**

**Fig. 4:**
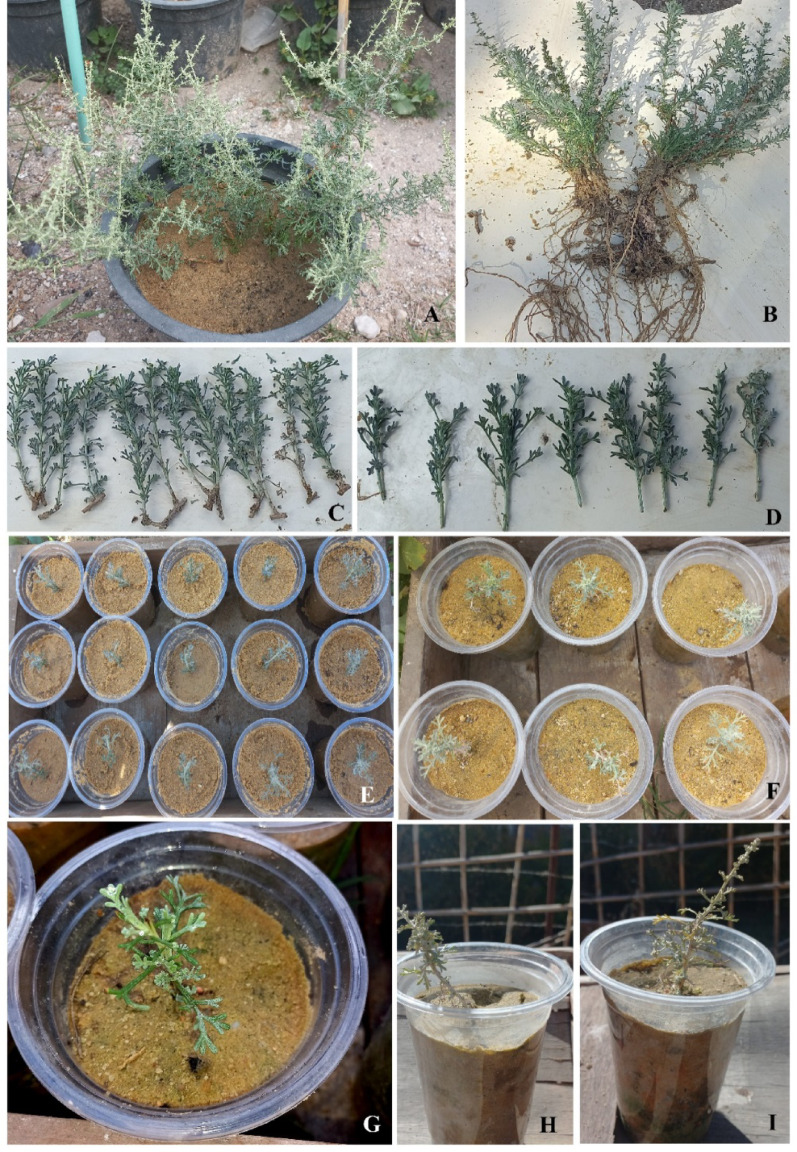
Vegetative propagation of *Artemisia herba-alba*. Well established plant growing from a seedling **(A)**, complete uprooted plants **(B)**, semi-woody cuttings **(C)**, herbaceous cuttings **(D)**, cuts after one month **(E)**, cuts after 5 months **(F)** & **(G)** complete growth and flowering **(H)** & **(I)**

The transplants used for vegetative propagation had been collected from the Western Mediterranean Coastal region of Egypt and domesticated for one year prior to propagation. It should be noted that the individuals used for vegetative propagation were not the same as those involved in the soil and water experimental trials. The vegetative propagation of *P. maritimum* was carried out by separating offsets from the parent bulb in January 2023 (Fig. 3), achieving a 100% success rate. In contrast, *A. herba-alba* was propagated using both herbaceous and semi-woody stem cuttings in sandy soil during two seasonal trials. The first propagation trial, conducted from December 2023 to February 2024, resulted in complete wilting of the cuttings within 37 days. However, the second trial, carried out from March to September 2024, demonstrated improved outcomes, with the development of new leaves and an increase in plant height. Initiating vegetative propagation during the spring season yielded an overall success rate of approximately 67%, comprising 40% success from herbaceous cuttings and 27% from semi-woody cuttings (Fig. 4).

## Discussion

Natural landscaping, which utilizes native plants, would enhance the sustainable use of water resources. In the Mediterranean region, xeriscaping has become very feasible due to the scarcity of water and the need to preserve it through sustainable landscaping practices [24]. By utilizing drought-tolerant plants and efficient irrigation techniques, xeriscaping provides a sustainable substitute for conventional landscaping [25]. Incorporating stress-tolerant ornamental plants into urban landscapes is essential for creating sustainable and resilient cities. These species withstand climate and urban stresses, enhance ecosystem services, support biodiversity, and improve human well-being, making them key to future urban planning strategies [26]. Native psammophilous plants, well-adapted to extreme conditions, were also promoted as sustainable ornamental options in a beach project in Liguria, Italy. Approximately 20 native species were selected and propagated using sustainable horticultural practices for application in xerogardening and urban landscaping [27]. Our study provides recommendations that may contribute to plant species conservation and promote the development of varied landscapes by determining the possible application of a native wild plant in sustainable landscape design approaches via the use of different irrigation treatments. Canga [4], also recommended the utilization of wild plants in all forms of landscape architecture to preserve the sustainability of the natural landscape. Tribulato et al. [28] indicated that techniques for determining water regimes can be compatible with both the preservation of ornamental quality and a constrained water supply. Álvarez et al. [29] also suggested gardening with modest deficit irrigation since it produced plants with great aesthetic value. The results of our study have demonstrated that it is possible to quantify the water-regime requirements of *Pancratium maritimum* and *Artemisia herba-alba* sustainably. For *P. maritimum*, all growth parameters (ground cover, leaf area, number of leaves, and maximum leaf length) showed significant differences (P˂ 0.001) among the water regime treatments except for ground cover with notable significant variations between the two soil types in all growth parameters except for leaf area and maximum leaf length. Additionally, insignificant effects of the soil type × water regime interaction on growth parameters are observed (*P* > 0.05). The data of our study recommended using water regime W2 using sandy soil S5 (W2S5) as it showed a considerable value of growth parameters and was not significantly different from the highest values for each studied growth parameter. In conclusion, the optimal irrigation procedure for *P. maritimum* landscaping should provide the greatest growth with an appropriate amount of water while maintaining the plant’s aesthetic value, which was expressed by treatment W2S5.

For *A. herba-alba*, the studied growth parameters, ground cover and plant height, showed significant differences (P˂ 0.001) among the water regime treatments with notable significant variations between the two soil types in all growth parameters. The soil type × water regime interaction also has a substantial significant impact (P˂ 0.05) on growth parameters. The data of our study recommended using water regime W2 using native soil S2 (W2S2), as it showed the highest growth in all studied growth parameters. Even though W2S2 consumes a little more water (88 ± 10 L/month/m^2^) as compared to W3 in both soil types, its highest mean values, minimal effort, and relatively low water consumption compared to other treatments may render it a more favorable option.

Additionally, previous studies such as Biella et al. [30] highlight that plant propagation is a critical step in successful biodiversity-focused urban greening. This process involves cultivating and increasing the number of plants, especially native species, to enhance habitat diversity and support various urban ecosystems. Vegetative propagation by bulb of *P. maritimum* in our study showed a success rate of 100%; similarly, *A. herba-alba* exhibited successful vegetative propagation by stem cuttings, both herbaceous and semi-woody. The propagation success rate was approximately 67%, maintaining the plant’s good appearance. Previous studies have indicated that seed propagation of both studied species is not recommended. For example, Paradiso et al. [31] indicated that *P. maritimum* could be propagated from seeds, but it takes at least five years to produce flowering plants. Furthermore, Chiboub et al. [32] suggested that the most effective and promising method for *A. herba-alba* propagation is the application of cuttings because gathering seeds from individuals of *A. herba-alba* for germination is a very difficult process. Our work and the similar study by Chiboub et al. [32] showed that this difficulty arises from the tiny size of seeds, making them difficult to detect.

It is also affected by many variables, such as the year-round climate, the seeds’ stage of maturity, the storage environment, and the length of storage. But it is quicker to get a new seedling via cuttings. Chiboub et al. [32] found that the cuttings from the seventh day had begun to emit roots, indicating that the plant is ready for field transplantation, and recommended propagating *A. herba-alba* asexually, specifically utilizing herbaceous apical cuttings that were gathered in March. Based on our findings, it is recommended to initiate vegetative propagation of *A. herba alba* in the spring season using sandy soil, as it yields a higher success rate for plant development as compared to the winter season. Of the total (67%) success rate, 40% came from herbaceous cuttings. This might indicate that the herbaceous cuttings of *A. herba-alba* are more effective for propagation under suitable conditions.

Choosing native plant species that can tolerate or adapt to the stress of water shortage may be an efficient way to improve landscaping, especially in semi-arid areas. The findings of Doll et al. [33] suggest that park managers retain significant flexibility to implement diverse groundcover designs that still achieve near-optimal social and environmental outcomes. Notably, parks with at least 40% native vegetation groundcover enhance public utility while promoting water conservation [33]. Mircea et al. [24] reported that *P. maritimum* is suited to provide beauty to the landscape without posing an invasive threat. Louhaichi et al. [34] reported that *A. herba-alba* had a great ability to withstand drought, making it a great choice for locations with low to moderate soil moisture levels and salinity. Our study revealed that both *P. maritimum* and *A. herba-alba* had effective strategies to mitigate the negative impacts of drought stress. Besides, the present study’s findings may substantially improve water valorization by using native plants in landscaping, especially in arid and semi-arid regions.

## Conclusion

The present study represents a preliminary investigation into the potential for large-scale application, aiming to minimize water usage while preserving the aesthetic quality of the plants. The findings of our study highlight the significant potential of native desert plants, particularly *Artemisia herba-alba* and *Pancratium maritimum*, for sustainable landscape design in arid and semi-arid areas. Both species demonstrate a strong ability to adapt to various soil types and water regimes, showcasing their effectiveness in conserving water while maintaining growth and aesthetic value. We identified the most effective irrigation techniques that maximize plant growth while minimizing water usage through careful assessment of different water-regime treatments. Additionally, the successful vegetative propagation of these plants illustrates their suitability for long-term use in landscaping projects. Overall, the findings indicate that integrating these native desert plants into landscape design offers a sustainable solution for addressing water scarcity and promoting biodiversity in challenging environments. The results can guide future landscape designs that prioritize both aesthetic value and ecological sustainability.

## Data Availability

All data have been provided with the manuscript.
